# Bi-directional high speed domain wall motion in perpendicular magnetic anisotropy Co/Pt double stack structures

**DOI:** 10.1038/s41598-017-05409-7

**Published:** 2017-07-10

**Authors:** P. Sethi, S. Krishnia, W. L. Gan, F. N. Kholid, F. N. Tan, R. Maddu, W. S. Lew

**Affiliations:** 1 School of Physical and Mathematical Sciences, Nanyang Technological University, 21 Nanyang Link, Singapore, 637371 Singapore; 2 Department of Physics, University of Cambridge, Cambridge, United Kingdom

## Abstract

We report bi-directional domain wall (DW) motion along and against current flow direction in Co/Pt double stack wires with Ta capping. The bi-directionality is achieved by application of hard-axis magnetic field favoring and opposing the Dzyloshinskii-Moriya interaction (DMI), respectively. The speed obtained is enhanced when the hard-axis field favors the DMI and is along the current flow direction. Co/Pt double stack is a modification proposed for the high spin-orbit torque strength Pt/Co/Ta stack, to improve its thermal stability and perpendicular magnetic anisotropy (PMA). The velocity obtained reduces with increase in Pt spacer thickness due to reduction in DMI and enhances on increasing the Ta capping thickness due to higher SOT strength. The velocity obtained is as high as 530 m/s at a reasonable current density of 1 × 10^12^ A/m^2^ for device applications. The low anisotropy of the device coupled with the application of hard-axis field aids the velocity enhancement by preventing Walker breakdown.

## Introduction

Non-volatility, low operating power and high endurance are some of the important features of domain wall (DW) based memory and logic devices^1–5^. Current induced domain wall motion (CIDWM) has generated a lot of research interest with the advent of spin-transfer torque (STT) technique^6, 7^. Recently, a more efficient method of DW propagation that is based on spin-orbit torque (SOT) originating at heavy-metal and ferromagnetic (FM) layer interface has become prominent^8–11^. Reports indicate that Néel DWs, stabilized via an anti-symmetric exchange interaction namely Dzyaloshinskii-Moriya interaction (DMI)^12^, can be driven by SOT even along the current flow direction at high speeds^13–15^. Very high DW velocity, *e.g*. 750 m/s was reported in magnetic layers with perpendicular magnetic anisotropy (PMA) coupled with synthetic anti-ferromagnets^16^. DW speed of 400 m/s was reported in Pt/Co/AlO_x_ PMA nanowires on account of Rashba effect^9^. However, the current density required in the above studies was in excess of 3 × 10^12^ A/m^2^. Low current alternative was proposed by Metaxas *et al*.^17^, where vertical current injection was used in a magnetic tunnel junction to drive the DW in the free layer. However, this method requires more complex device processing and the distance propagated by DW is confined to nanometres. There is a need of exploring DW dynamics in other multi-layers possessing large SOT strength. One such system that has not been explored extensively for studying DW dynamics is Pt/Co/Ta where the heavy-metal layers provide the SOT and DMI strength. Pt/Co/Ta stack has been reported to exhibit enhanced SOT on account of opposite signs of spin Hall angles of Ta and Pt acting on top and bottom interfaces. However, it suffers from diffusion of Ta into Co which may lead to degradation of PMA^18^. Recently, we proposed and estimated the SOT strength of multilayer stack, namely Co/Pt double stack, with four interfaces namely Pt/Co/Pt and Pt/Co/Ta possessing higher thermal stability and stronger PMA than single Pt/Co/Ta stack^19^. In this letter, DW dynamics have been investigated in the Co/Pt double stack by fabricating Hall cross structures. The hard-axis magnetic field was also applied to either support or oppose the effective field due to DMI. An enhancement in DW velocity was observed on increasing the Ta capping thickness and reducing the Pt spacer thickness along the current flow direction.

## Results and Discussion

In this study, CIDWM was investigated using anomalous Hall effect (AHE)^20, 21^ and Kerr magneto-optical microscopy in Hall cross structures. Thin films were deposited on silicon wafers that coated with silicon dioxide (SiO_2_) using magnetron sputtering technique at a base pressure of 2 × 10^−8^ Torr. The structure of the films was of the form SiO_2_/Ta (3)/Pt (3)/Co (0.7)/Pt (0.5)/Co (0.7)/Ta (1), where thicknesses are in nm. A Pt dusting layer of thickness 1 nm was deposited on top to prevent oxidation of the capping Ta layer. Since the Pt dusting layer is not in direct contact with the ferromagnetic layer, Co, it would not affect the SOT strength and DW dynamics. The saturation magnetization, M_S_ was obtained as 700 emu/cc using alternating gradient force magnetometry (AGFM). The out-of-plane anisotropy constant was estimated to be 1.4 × 10^6^ ergs/cc (measurements are shown in Supplementary Information S1). A 1.5 µm wide and 30 µm long Hall cross structure was fabricated using electron beam lithography and argon ion-milling techniques. The structure exhibits PMA and current induced magnetization switching indicating the existence of SOT as demonstrated previously^19^.

**Figure 1:**
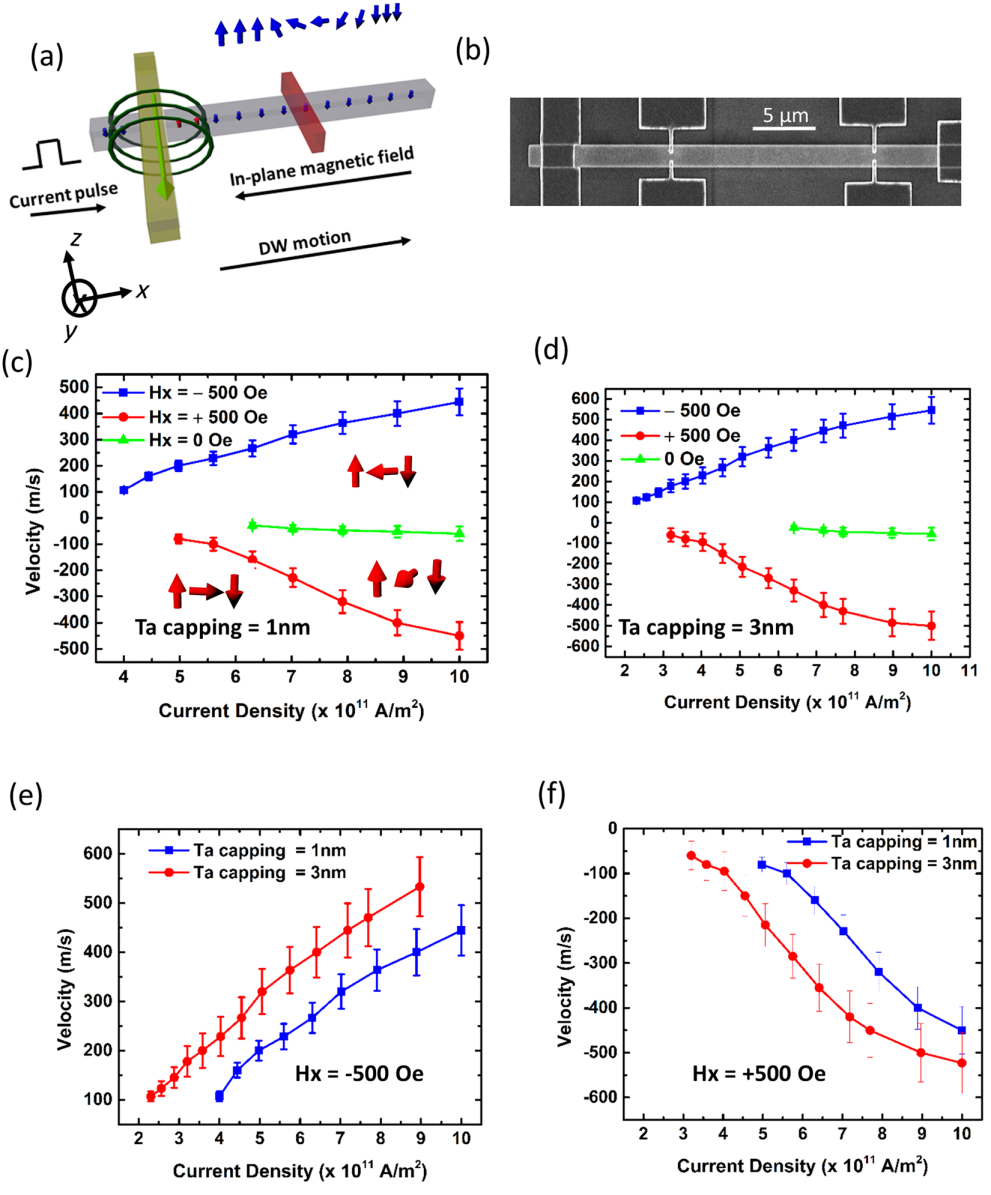
(**a**) Schematic depicting the setup for domain wall (DW) injection and driving measurement. (**b**) SEM image of the wire with non-magnetic Hall probes for DW detection. DW velocity versus current density under a constant in-plane magnetic field along + *x* and −*x*-directions for Ta capping with thickness (**c**) 1 nm, and (**d**) 3 nm. Comparison of DW velocity for Ta capping thicknesses 1 nm and 3 nm; magnetic field was applied along the (**e**) −*x*-direction, and (**f**) + *x*-direction.

DW injection was carried out using local Oersted field approach by passing a pulse current through an electrical strip-line^22^. Figure 1a illustrates the DW injection and driving setup. An up-down DW was nucleated by applying a current pulse along a metallic π-shaped strip-line^23^. A constant in-plane magnetic field was applied using a bar magnet along the −*x*-direction to create a left-handed chirality Néel wall. The DW was driven by an application of current pulse and detected by a drop in R_Hall_ at the Hall probe. Figure 1b shows SEM image of the fabricated device. A Ta Hall bar of width 200 nm was also patterned to detect the position of the DW. The Hall probe was made non-magnetic to prevent DW pinning. Figure 1b shows two Hall probes, but in our measurements first Hall probe was used for detection. The distance between the Hall probe and metallic strip-line was kept as 8 µm. The DW velocity was determined by noting the current pulse width for the DW to reach the Hall probe. This was validated by Kerr microscopy imaging.

Figure 1c shows the plot of DW velocity with change in the applied current density under the application of fixed in-plane magnetic field of strength 500 Oe. When the applied field is in the −*x*-direction, the up-down DW propagates along the current flow direction. This is consistent with previous reports which suggest that left-handed Néel wall propagates along the current flow direction if the underlayer has a positive spin Hall angle^13, 24, 25^. The minimum current density for DW propagation was found to be 3.5 × 10^11^ A/m^2^. The DW velocity is found to increase linearly with the applied current density and reached a value of 440 m/s at an applied current density of 1 × 10^12^ A/m^2^. The current density was limited to such value to prevent random domain nucleations and joule heating effect^22^. When the magnetic field direction was reversed, the DW was found to propagate against the current flow direction. The minimum current density for finite velocity increased to 5 × 10^11^ A/m^2^. The field direction propelled DW to take a right-handed Néel chirality, hence the direction of DW motion also reversed^13^. It was observed that at lower current densities, DW velocity along the current flow direction was higher than that along the electron flow direction. This indicates the existence of a small DMI that favours left-handed Néel chirality, which opposes the in-plane magnetic field. When the direction of the field is reversed the DW velocity changes sign, indicating the DMI field is less than the applied field strength. Against the current flow direction, STT is also expected to contribute to the DW velocity, however, such contribution is expected to be less at lower current densities ( < 7 × 10^11^ A/m^2^)^26–28^. The measurement results reveal that the SOT is able to compensate the STT effect when the DW propagates along the current flow direction. When the external field was removed, the DW was found to propagate in the electron flow direction, with minimum current density needed for propagation in excess of 6 × 10^11^ A/m^2^. This indicates the existence of Bloch DW being driven primarily by STT^14^. The magnitude of velocity was found to be much lower than that in the presence of field with maximum velocity around 60 m/s at a current density of 1 × 10^12^ A/m^2^. We also performed micromagnetic simulations to test the direction of motion of DWs and their structure in the presence of an in-plane field which assists and opposes the motion along the current flow direction. The results are shown in Supplementary Information S2.

To investigate the effect of Ta capping thickness, the Ta thickness was increased to 3 nm, keeping the other layer thicknesses intact including the Pt dusting layer of 1 nm, and the DW velocity was measured. Figure 1d shows the linear relationship of velocity with the applied current density, which has similar behaviour to that shown in Fig. 1c. Figure 1e shows the comparison between DW velocities with 1 nm and 3 nm Ta thicknesses, respectively, when the applied in-plane field is in the −*x*-direction. The plots indicate that the velocity increases nearly 1.5 times when the thickness is raised by 3 times. Increasing the Ta thickness enhances the current flow in the heavy metal layer thereby generating more spin current leading to larger SOT on the ferromagnetic layer, shown previously by us^19^. However, the efficiency of the heavy metal to separate the spin components would tend to reduce when the thickness is increased beyond a certain limit on account of finite spin diffusion length^29, 30^. Thus, the thickness of Ta was limited to 3 nm, in our system. Figure 1f shows the comparison of velocities when the field direction is reversed. In this case also the velocity enhances with increase in the Ta capping thickness. The velocity obtained by increasing the Ta layer thickness is as high as 530 m/s which is comparatively higher than reported previously at the applied current density of 1 × 10^12^ A/m^2^. The above electrical measurements were repeated for down-up DW and the results are shown in Supplementary Information S3.

**Figure 2:**
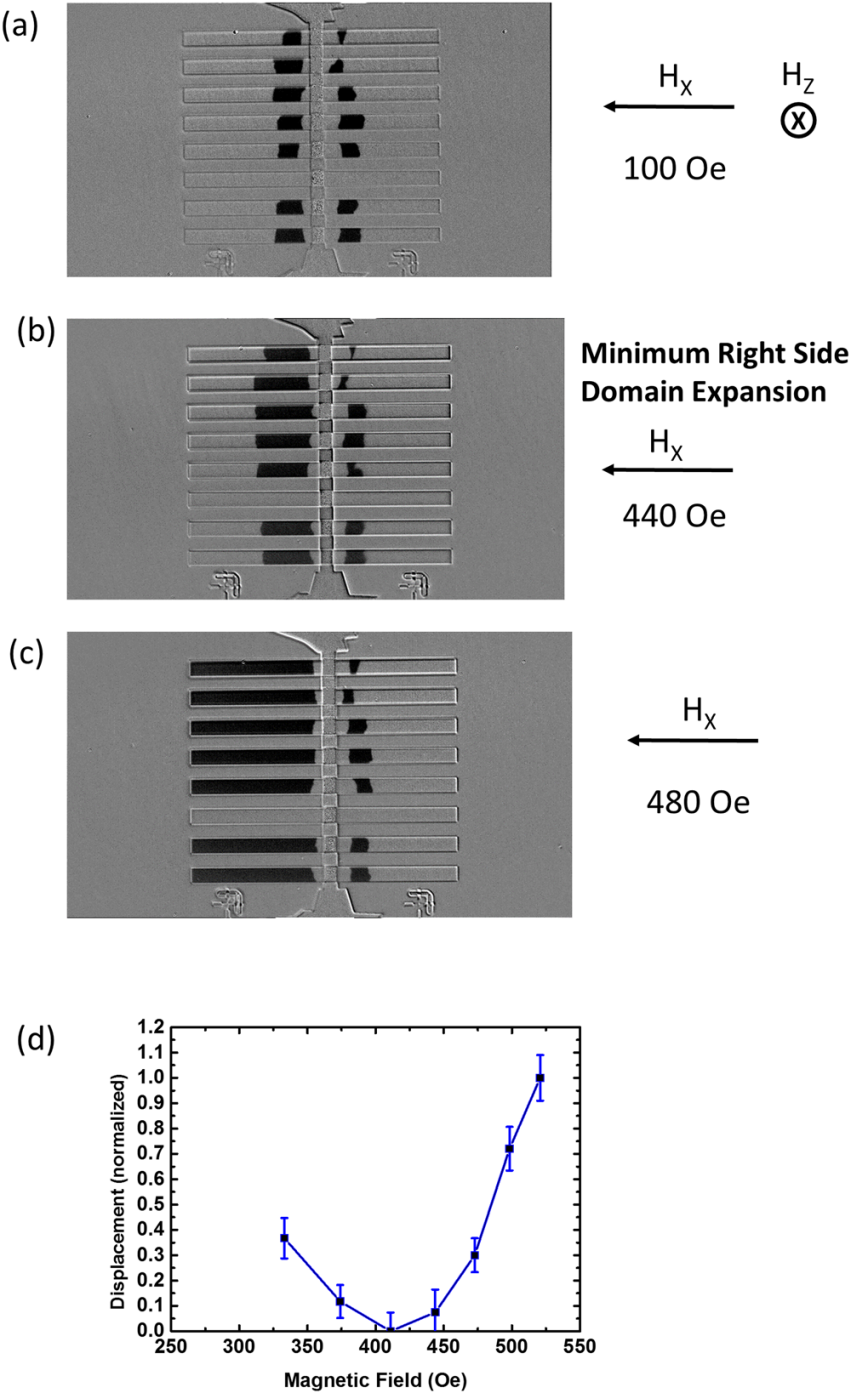
(**a**–**c**) Kerr imaging to show DW creep in the presence of out-of-plane and in-plane magnetic field. The in-plane field opposes DMI field for DW propagating towards right and minimum displacement occurs at field equal to DMI field. Here H_DMI_ = 440 Oe. (**d**) Domain wall displacement as a function of in-plane magnetic field.

DMI field was computed using DW creep method^31, 32^. Thin film samples of the original composition SiO_2_/Ta (3)/Pt (3)/Co (0.7)/Pt (0.5)/Co (0.7)/Ta (1), were fabricated into 5 µm wide micron strips. An injection line was patterned in a second lithography step towards the middle of the wires. The width of the wires was chosen large to get a good contrast and image in the Kerr microscopy measurements. Figure 2a–c shows the Kerr images of the device when after injecting two domain walls in the middle, out-of-plane field was swept to drive DWs in opposite directions. An in-plane field was also applied along the − *x*-direction to speed up or slow down the DWs. The sequence of Kerr imaging is as follows: first the current is injected in the stripline to nucleate the DWs, which are allowed to expand slightly in the horizontal direction with small out-of-plane field. Then a reference image is taken. After this, the out-of-plane field is applied along with an in-plane field to drive the DWs in opposite directions. The image is again taken and subtracted from the reference. This the reason that in the images, the region next to the stripline appears without any domain (or gray in color as the background). It is observed that the domains are not perfect since the widths of the nanowires are large and there is a possibility of multiple domans or pinning sites deforming the domains. The in-plane field values are indicated in the images. The dark contrast represents down magnetization. Since the DWs have left hand Neel chirality, the DWs on the left of the stripline would always propagate faster since the in-plane field is assisting it. The DWs on the right of the stripline would propagate slower since the in-plane field would oppose field due to DMI or H_DMI_. For applied in-plane field much less than H_DMI_, the DWs propagate at reasonable speed. As the field is increased towards the H_DMI_, the speed or the displacement as observed in the Kerr images start to drop. The speed was found to be minimum at around 400 Oe of applied in-plane field. When the applied field increases beyond H_DMI_, it overcomes the DMI field and the DW is able to expand again at a faster rate. Figure 2d shows the plot of the displacement (normalized) with respect to the applied in-plane field. The plot shows a minimum at about 420 Oe. This can be considered as a rough estimate of H_DMI_. This shows that the DMI field is less than the applied field of 500 Oe in the experiments and corroborates with our DW velocity measurements. In the ideal case both the domain walls would be nucleated on one side of the stripline due to the symmetry of the Oersted field. We believe that in practical devices, due to the temperature fluctuations and non-homogenity of the field and the device geometry, DWs nucleation could happen at the film underneath the stripline, which could then displace on the other side of it. It is worth noting that even if the DWs are nucleated on one side of the stripline they would move away from each other as soon as a small out-of-plane field is applied, together with the thermal effects.

**Figure 3:**
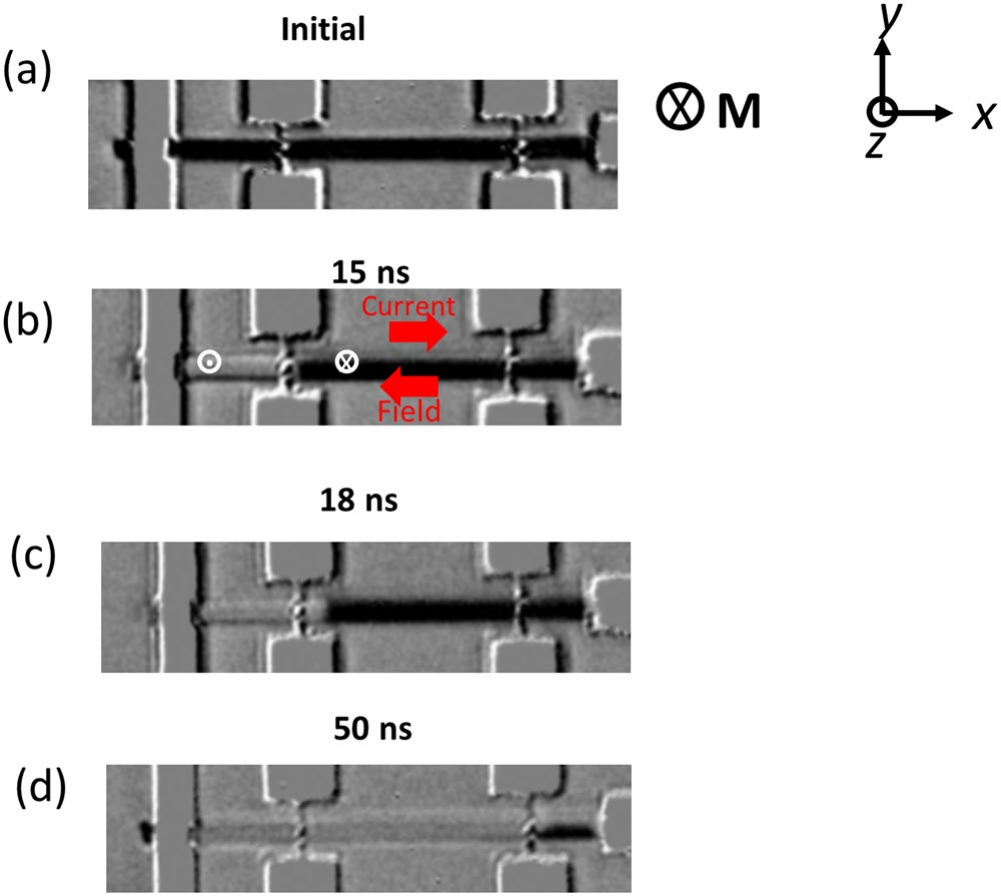
Direct observation of SOT-driven DW motion in Ta/Pt/Co/Pt/Co/Ta structure using Kerr imaging technique. (**a**) Initial magnetization state in −*z*-direction. (**b**) DW motion after the application of a 15 ns pulse, the DW propagates 8 µm distance and reaches the first Hall probe (**c**) application of 18 ns pulse, DW depins from the Hall probe and propagates a distance of 10 µm and (**d**) application of 50 ns pulse, DW reaches second Hall probe propagating a distance of 25 µm.

The electrical measurements were supported by direct observation of DW motion through magneto-optical Kerr microscopy imaging technique. Figure 3a shows the Kerr image of the device with dark contrast indicating magnetization in the −*z*-direction. After DW injection, a current pulse of 1 × 10^12^ A/m^2^ and width 15 ns was applied to drive the DW. Kerr image in Fig. 3b shows that the DW reaches the Hall probe after propagating a distance of 8 μm in 15 ns, which gives a DW velocity of 530 m/s. Similar measurement was repeated with a pulse width of 18 ns, and the Kerr image in Fig. 3c shows DW displaced past the first Hall probe propagating a distance of 10 μm. When a 50 ns pulse was applied the DW moved past the second Hall probe propagating a distance of 25 μm, as shown in Fig. 3d.

**Figure 4:**
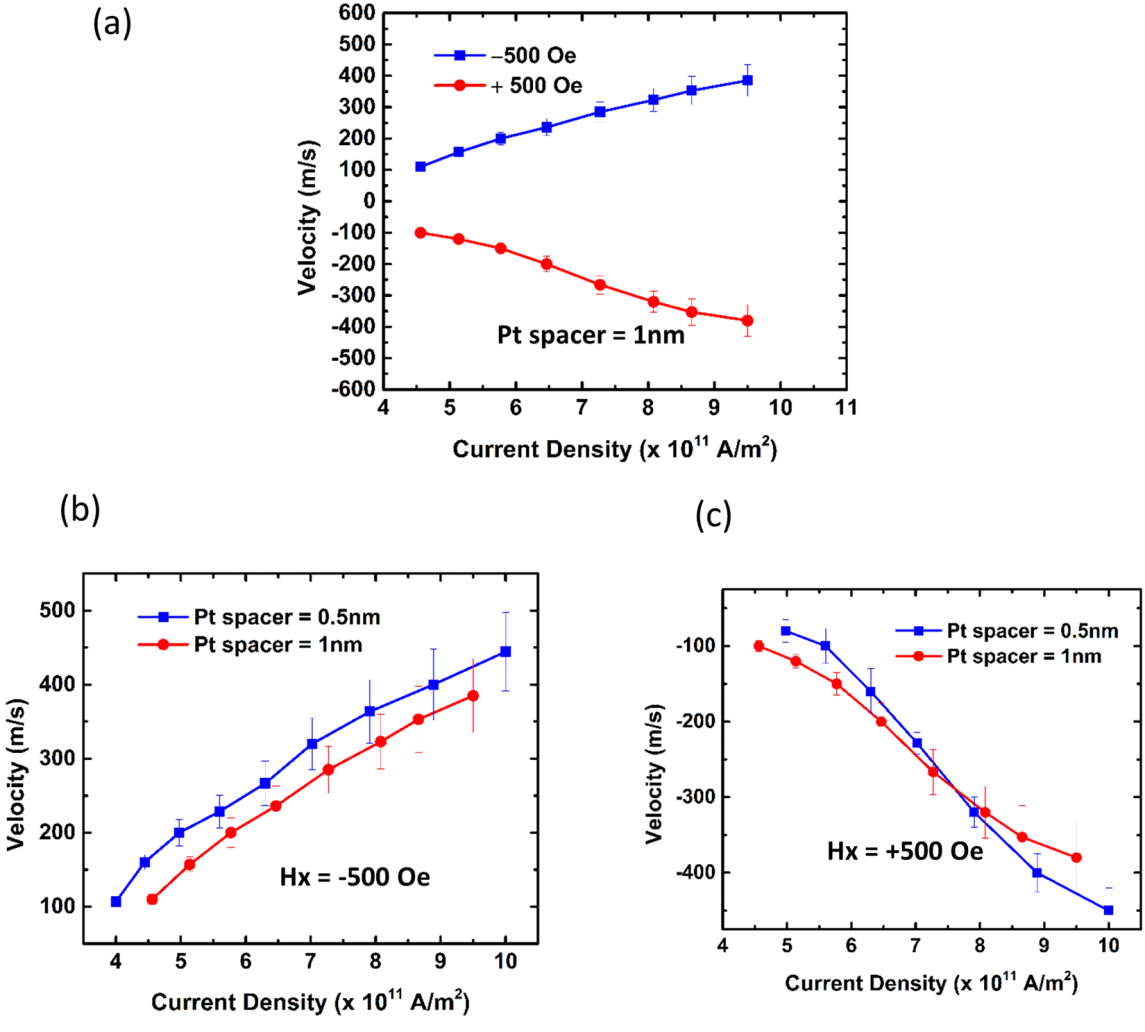
(**a**) DW velocity versus current density in the presence of a constant in-plane magnetic field in + *x* and −*x*-directions for 1-nm-thick Pt spacer; plot symmetry across x-axis reveals low DMI strength. DW velocity for Pt spacer with thicknesses 0.5 nm and 1 nm when a magnetic field was applied along (**b**) −*x*-direction, and (**c**) + *x*-direction. Comparison indicates increasing Pt spacer thickness reduces velocity.

To examine the effect of Pt spacer layer on domain wall velocity, Pt thickness was increased to 1 nm while keeping Ta capping layer thickness fixed at 1 nm. Figure 4a summarizes the results for DW velocity at different applied current densities. The plots appear symmetric with respect to the *x*-axis. This indicates that the magnitude of the velocity along and against current flow direction is same when the applied field direction is reversed. Thus this stack has lesser DMI strength than the one with lesser Pt spacer thickness. This is expected as the DMI from underlayer and spacer Pt would tend to compensate each other if the layer thicknesses are closer^33^. In the current flow direction, the SOT would act on the DW if the DMI strength can sustain a chiral Néel DW, where the demagnetization energy tends to favor a Bloch DW formation. In our sample stack, the presence of Néel DWs is realized by the application of external in-plane field. When the direction of the external field is reversed, the internal field generated by the DMI would be in the opposite direction. However, due to the low DMI strength, the counter effect is negligible hence there is no reduction in the DW velocity. Moreover, the DW velocity is expected to increase against the current flow direction due to STT, though it is not directly observed from our experimental results. This implies that all the forces are balanced. Thus, the increase in DW velocity due to the STT is compensated by the small DMI that favors left handed Néel DW as well as motion along the current flow direction.

Figure 4b shows a comparison of the velocities between devices with Pt thicknesses 0.5 nm and 1 nm respectively, when the applied field is in the −*x*-direction. Increasing the Pt spacer thickness would have two effects: firstly, it would lower the DMI strength; secondly, the SOT would also reduce from the bottom half of the stack, reason being that Pt/Co and Co/Pt interfaces are competing with each other with regard to the SOT. However, the SOT from the top half of the stack, which is due to the sum of Pt/Co and Co/Ta interfaces, would increase on increasing the Pt thickness as more spin current would induce torque on the upper Co layer. Spin Hall angle measurements were reported previously by us^19^. The net influence of the above contributions would affect the final DW velocity. The reduction in velocity is not very significant, which could be due to opposite effects from the bottom and top interface with regards to the SOT contribution. Figure 4c shows the comparison of the velocities when the direction of the field is reversed. When the current density is low, the device with Pt spacer thickness as 1 nm has larger velocity than the device with Pt spacer thickness as 0.5 nm. This result is counterintuitive, however, we note that the device with larger Pt spacer thickness has little effect from DMI, hence, when the field is in the + *x*-direction, there is little opposition to the applied field. Whereas, the device with less Pt spacer thickness would experience more resistance to the applied field on account of larger DMI strength. As the current density increases there is a cross-over and beyond current density of 8 × 10^11^ A/m^2^, the DW velocity for the device with Pt spacer thickness 0.5 nm is larger than the device with spacer thickness 1 nm. This indicates that at larger current density the effect of SOT dominates and it appears that the bottom Pt/Co/Pt stack contribution increases the DW velocity for device with lesser Pt spacer thickness, even though the SOT contribution from the top Pt/Co/Ta stack would be lower. The above electrical measurements were repeated for down-up DW (the results are shown in Supplementary Information S3). The results for DMI field measurement are shown in the Supplementary Information S4. We have also performed the XPS measurements for our stack, where we observe no trace of TaOx or Co oxidation and very little intermixing between Ta and the middle Co layer. The results and discussion on comparison with single stack Pt/Co/Ta are provided in the Supplementary Information S5.

Although the change in SHA could account for the trend in the velocity with thickness variation, the magnitude obtained are much less to account for the high velocities observed. The stray field from the bar magnet was measured and it was found to be 5–6 Oe, which is too low to drive the DW. The simulated stray field of the bar magnet is shown in the Supplementary Information S6. Moreover, the direction of the DW motion could be reversed on switching the current flow direction, which indicates negligible out-of-plane stray field. The low out-of-plane anisotropy of our device coupled with the application of hard-axis field to increase Walker breakdown field^9^ help to contribute to the velocity enhancement^34^.

In summary, high speed domain wall motion with velocity greater than 500 m/s at an applied current density of 1 × 10^12^ A/m^2^ was observed in Co/Pt double stack wires. An in-plane field stabilized chiral Néel walls are propagated along or against the current flow direction depending on the chirality, which can be reversed by changing the polarity of the applied field. The hard-axis field is expected to increase the Walker breakdown field^9, 35^, which could also potentially contribute to the velocity increment. The DW velocity increases with larger Ta capping layer thickness on account of stronger SOT strength, and the velocity decreases with larger Pt spacer thickness on account of weaker DMI and SOT strengths from the bottom Pt/Co/Pt interface. Thus, double layer Co/Pt stacks having enhanced thermal stability potentially leads towards new avenues of research in terms of high speed logic and memory applications.

## Methods

### Device Fabrication

The nanostructures were fabricated on Si/SiO_2_ (300 nm) substrates. The substrates were cleaned with acetone and rinsed with iso-propyl alcohol for 20 mins each under ultrasonic agitation, and then blow dried with N_2_. A thin film stack of Ta/Pt/Co/Pt/Co/Ta/Pt was deposited using ultra high vacuum magnetron sputtering technique. The wafers were then spin-coated with negative resist and the nanostructured patterns were written by using electron beam lithography. The pattern transfers were completed using Ar ion milling technique. The negative resist was subsequently removed by using oxygen plasma stripper. To overlay with contacts, a second exposure was done after coating the sample with positive photo-resist. The Ta/Cu contact was deposited using sputtering and capped with Au to prevent oxidation. The photo-resist was removed by lift-off technique.

### Electrical measurements

Domain wall (DW) injection and driving was carried out using 40 GHz Cascade Microtech probe station. Programmable pulse generator (Picosecond 10300B) was connected to a radio-frequency probe for injecting and driving the DW. Keithley voltmeter (2000) and sourcemeter (2400) were employed for reading the Hall signals. To nucleate a DW, the wire was first saturated with large out-of-plane field and then a voltage pulse was applied to the current carrying strip-line. To drive the DW, this pulse was applied across the wire and the pulse width corresponding to drop in Hall signal was recorded. This served as a time of flight method to determine the DW velocity.

### Kerr microscopy

In-house developed Kerr microscopy in polar configuration was employed for detecting the presence of DWs. The magnetized sample injected with the DW was subjected to a plane polarized light. The light reflected from the sample was detected by the analyser placed in a near cross position with the polarizer. The Halogen lamp was used as the light source. The reflected light was captured by a camera and the image was processed in Matlab to obtain the final Kerr image. Two images were taken, one before the injection and one after. The two images were subtracted to obtain the final image.

## Electronic supplementary material

Supplementary Information

## References

[1] Allwood DA (2005). Magnetic domain-wall logic. Science.

[2] Parkin SS, Hayashi M, Thomas L (2008). Magnetic domain-wall racetrack memory. Science.

[3] Goolaup S, Ramu M, Murapaka C, Lew WS (2015). Transverse domain wall profile for spin logic applications. Sci Rep.

[4] Murapaka C, Sethi P, Goolaup S, Lew WS (2016). Reconfigurable logic via gate controlled domain wall trajectory in magnetic network structure. Sci Rep.

[5] Sethi P (2016). Direct observation of deterministic domain wall trajectory in magnetic network structures. Sci Rep.

[6] Berger L (1996). Emission of spin waves by a magnetic multilayer traversed by a current. Phys Rev B.

[7] Slonczewski JC (1996). Current-driven excitation of magnetic multilayers. J Magn Magn Mater.

[8] Miron IM (2011). Perpendicular switching of a single ferromagnetic layer induced by in-plane current injection. Nature.

[9] Miron IM (2011). Fast current-induced domain-wall motion controlled by the Rashba effect. Nat Mater.

[10] Liu L (2012). Spin-torque switching with the giant spin Hall effect of tantalum. Science.

[11] Haazen PP (2013). Domain wall depinning governed by the spin Hall effect. Nat Mater.

[12] Moriya T (1960). Anisotropic Superexchange Interaction and Weak Ferromagnetism. Phys Rev.

[13] Emori S, Bauer U, Ahn SM, Martinez E, Beach GS (2013). Current-driven dynamics of chiral ferromagnetic domain walls. Nat Mater.

[14] Khvalkovskiy, A. V. *et al*. Matching domain-wall configuration and spin-orbit torques for efficient domain-wall motion. *Phys Rev B* **87**, doi:10.1103/Physrevb.87.020402 (2013).

[15] Ryu KS, Thomas L, Yang SH, Parkin S (2013). Chiral spin torque at magnetic domain walls. Nat Nanotechnol.

[16] Yang SH, Ryu KS, Parkin S (2015). Domain-wall velocities of up to 750 m s(-1) driven by exchange-coupling torque in synthetic antiferromagnets. Nat Nanotechnol.

[17] Metaxas, P. J. *et al*. High domain wall velocities via spin transfer torque using vertical current injection. *Sci Rep-Uk* **3**, doi:10.1038/Srep01829 (2013). 10.1038/srep01829 PMC3653216 23670402

[18] Woo, S., Mann, M., Tan, A. J., Caretta, L. & Beach, G. S. D. Enhanced spin-orbit torques in Pt/Co/Ta heterostructures. *Appl Phys Lett* **105**, doi:10.1063/1.4902529 (2014).

[19] Sethi P, Krishnia S, Li SH, Lew WS (2017). Modulation of spin-orbit torque efficiency by thickness control of heavy metal layers in Co/Pt multilayers. J Magn Magn Mater.

[20] Yamanouchi M, Chiba D, Matsukura F, Ohno H (2004). Current-induced domain-wall switching in a ferromagnetic semiconductor structure. Nature.

[21] Ravelosona, D., Mangin, S., Katine, J. A., Fullerton, E. E. & Terris, B. D. Threshold currents to move domain walls in films with perpendicular anisotropy. *Appl Phys Lett* **90**, doi:10.1063/1.2450664 (2007).

[22] Sethi, P., Murapaka, C., Lim, G. J. & Lew, W. S. In-plane current induced domain wall nucleation and its stochasticity in perpendicular magnetic anisotropy Hall cross structures. *Appl Phys Lett* **107**, doi:10.1063/1.4935347 (2015).

[23] Zhang, S. F. *et al*. Highly Efficient Domain Wall Injection in Perpendicular Magnetic Anisotropy Nanowire. *Sci Rep-Uk* **6**, doi:10.1038/Srep24804 (2016). 10.1038/srep24804 PMC4838865 27098108

[24] Belmeguenai, M. *et al*. Interfacial Dzyaloshinskii-Moriya interaction in perpendicularly magnetized Pt/Co/AlOx ultrathin films measured by Brillouin light spectroscopy. *Phys Rev B* **91**, doi:10.1103/PhysRevB.91.180405 (2015).

[25] Boulle O (2016). Room-temperature chiral magnetic skyrmions in ultrathin magnetic nanostructures. Nat Nanotechnol.

[26] Tanigawa H (2009). Domain Wall Motion Induced by Electric Current in a Perpendicularly Magnetized Co/Ni Nano-Wire. Appl Phys Express.

[27] Koyama T (2011). Observation of the intrinsic pinning of a magnetic domain wall in a ferromagnetic nanowire. Nat Mater.

[28] Ueda K (2011). Current-Induced Magnetic Domain Wall Motion in Co/Ni Nanowire at Low Temperature. Appl Phys Express.

[29] Hahn, C. *et al*. Comparative measurements of inverse spin Hall effects and magnetoresistance in YIG/Pt and YIG/Ta. *Phys Rev B* **87**, doi:10.1103/Physrevb.87.174417 (2013).

[30] Allen, G., Manipatruni, S., Nikonov, D. E., Doczy, M. & Young, I. A. Experimental demonstration of the coexistence of spin Hall and Rashba effects in beta-tantalum/ferromagnet bilayers. *Phys Rev B* **91**, doi:10.1103/Physrevb.91.144412 (2015).

[31] Jue E (2016). Domain wall dynamics in ultrathin Pt/Co/AlOx microstrips under large combined magnetic fields. Phys Rev B.

[32] Je, S. G. *et al*. Asymmetric magnetic domain-wall motion by the Dzyaloshinskii-Moriya interaction. *Phys Rev B* **88**, doi:10.1103/Physrevb.88.214401 (2013).

[33] Ryu, K. S., Yang, S. H. & Parkin, S. Experimentally tunable chiral spin transfer torque in domain wall motion. *New J Phys* **18**, doi:10.1088/1367-2630/18/5/053027 (2016).

[34] Kim, D. H. *et al*. Maximizing domain-wall speed via magnetic anisotropy adjustment in Pt/Co/Pt films. *Appl Phys Lett* **104**, doi:10.1063/1.4871091 (2014).

[35] Bryan MT, Schrefl T, Atkinson D, Allwood DA (2008). Magnetic domain wall propagation in nanowires under transverse magnetic fields. J Appl Phys.

