# Supplementary material for: Bi-directional high speed domain wall motion in perpendicular magnetic anisotropy Co/Pt double stack structures
Source: Sci Rep. 2017 Jul 10;7:4964. doi: 10.1038/s41598-017-05409-7 (PMC5504067; doi:10.1038/s41598-017-05409-7)
Supplement: Supplementary file 1 — Supplementary Information [file 41598_2017_5409_MOESM1_ESM.pdf]

Bi-directional high speed domain wall motion in perpendicular magnetic anisotropy Co/Pt double stack structures

P. Sethi, S. Krishnia, W. L. Gan, F.N. Kholid[1]. F. N. Tan, R. Maddu and W. S. Lew*

School of Physical & Mathematical Sciences, Nanyang Technological University

21 Nanyang Link, Singapore 637371

SUPPLEMENTAL MATERIAL

Contents:

S1. Determination of anisotropy constant of thin film stack

S2. Effect of in-plane field on domain wall motion and structure

S3. Domain wall velocities: Down-Up chirality

S4. Estimation of DMI field for larger Pt spacer thickness

S5. X-ray photoelectron spectroscopy of Co/Pt double stack

S6. Stray field of permanent bar magnet

S7. Time of flight technique for domain wall velocity measurement

*Corresponding author: wensiang@ntu.edu.sg

[1]Current address: Department of Physics, University of Cambridge, United Kingdom

S1 Determination of anisotropy constant of thin film stack

The out-of-plane anisotropy constant, K_u was estimated from the relation, $K_u = M_S H_K/2$, where M_S is the saturation magnetization and H_K is the hard-aixs saturation field or the field at which the PMA sample saturates with the application of in-plane external field. Figure S1 (a) shows the M-H loops for original thin film sample having the composition Ta (3)/Pt (3)/Co (0.7)/Pt (0.5)/Co (0.7)/Ta (1). Figure S1 (b) shows the plots when Pt spacer thickness is increased from 0.5 nm to 1 nm keeping other thicknesses fixed. Figure S1 (c) shows the plots when Ta capping thickness is increased from 1 nm to 3 nm keeping Pt spacer thickness as 0.5 nm.

The plot in Figure S1 (a) is magnified to show clear square hysteresis of the out-of-plane M-H loop. The vertical line intersects the *x*-axis at the corresponding H_K values, which are 4000, 3000 and 3000 Oe respectively for the three thin films. The M_S for three thin films are respectively, 700, 820 and 900 emu/cc. The K_U for the three thin films are respectively, 1.4×10^6, 1.23×10^6 and 1.35×10^6 ergs/cc.

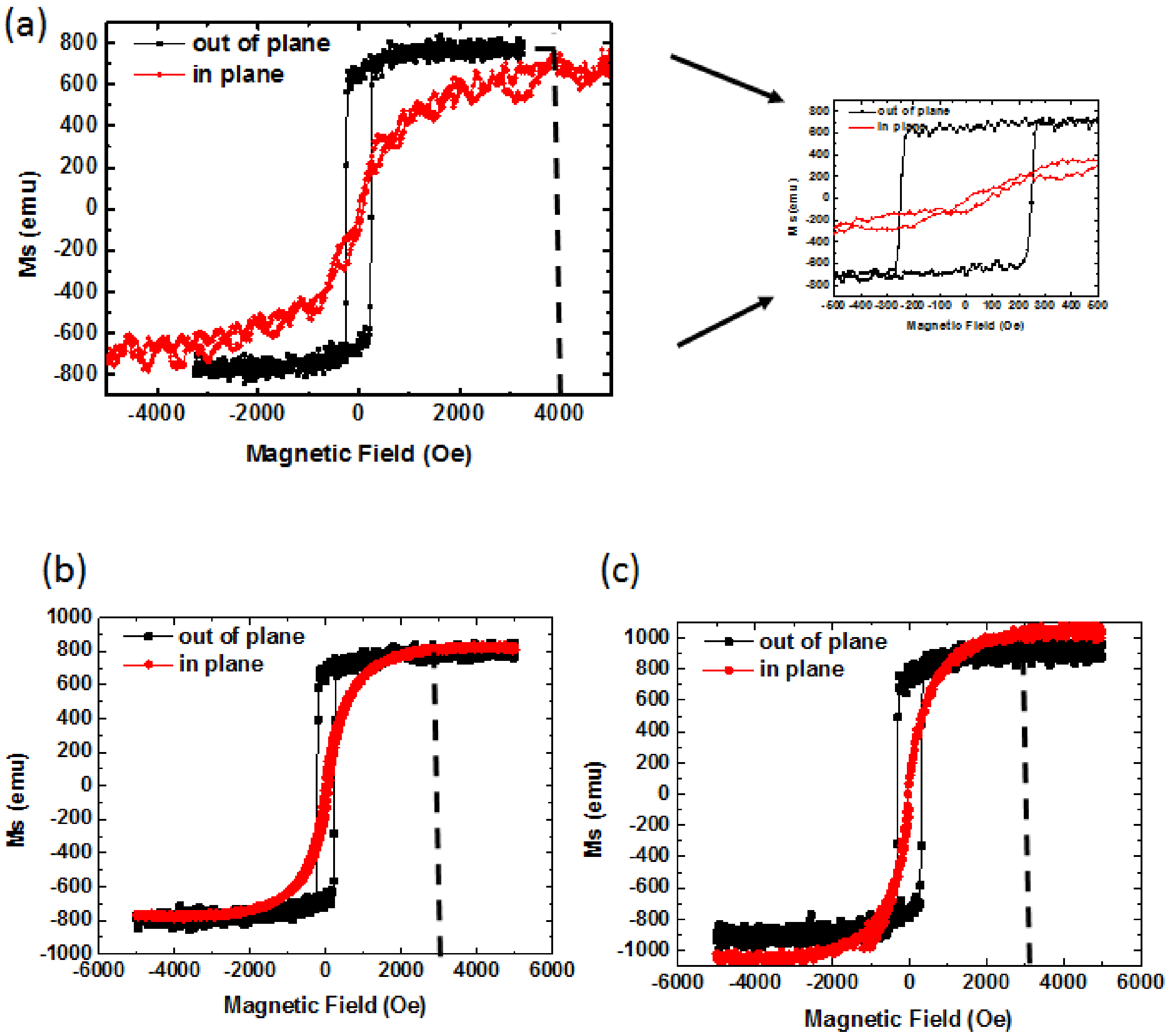

Figure S1. AGFM measurements of thin film stack for estimation of anisotropy constant. (a) Thin film stack Ta (3)/Pt (3)/Co (0.7)/Pt (0.5)/Co (0.7)/Ta (1). The inset shows a magnified view of the out-of-plane M-H loop to indicate square hysteresis (b) Pt spacer thickness increased, Ta (3)/Pt (3)/Co (0.7)/Pt (1)/Co (0.7)/Ta (1). (c) Ta thickness increased Ta (3)/Pt (3)/Co (0.7)/Pt (0.5)/Co (0.7)/Ta (3).

S2. Effect of in-plane field on domain wall motion and structure

In the presence of an in-plane field, the DW would be canted and have some tilt. It would have a Néel component. It would not be a perfect Bloch wall even if the width of the nanowire is large. If the DMI is present indeed the structure would be perfect Néel and in-plane field would either support or oppose the motion. Figure S2 shows the simulations performed using MuMag simulation tool[1,2].

Figure S2 (a)-(i) shows the initial simulated configuration at time t = 0, when a small Dzyaloshinskii-Moriya interaction (DMI) , D = 0.1 mJ/m^2 stablilizes a left handed Néel wall. A current is applied along the +*x*-direction. An in-plane field = 500 Oe was applied along the − *x*-direction to assist the left handed chirality. Figure S2 (a)-(ii) shows the final simulated configuration at a finite time. The DW propagates along the current flow direction and while in motion, the spins adopt a chirality which is partially Néel and partially Bloch.

Figure S2 (b)-(i) shows the initial simulated configuration at time t = 0, when a small Dzyaloshinskii-Moriya interaction (DMI) , D = 0.1 mJ/m^2 stablilizes a left handed Néel wall. A current is applied along the +*x*-direction. An in-plane field = 500 Oe is now applied along the +*x*-direction to oppose the left handed chirality. Figure S2 (b)-(ii) shows the final simulated configuration at a finite time. The DW propagates against the current flow direction as shown in our experiments. While in motion, the spins adopt a chirality which is Bloch, hence the motion is along the electron flow direction. In this case the in-plane field is able to overcome the DMI strength.

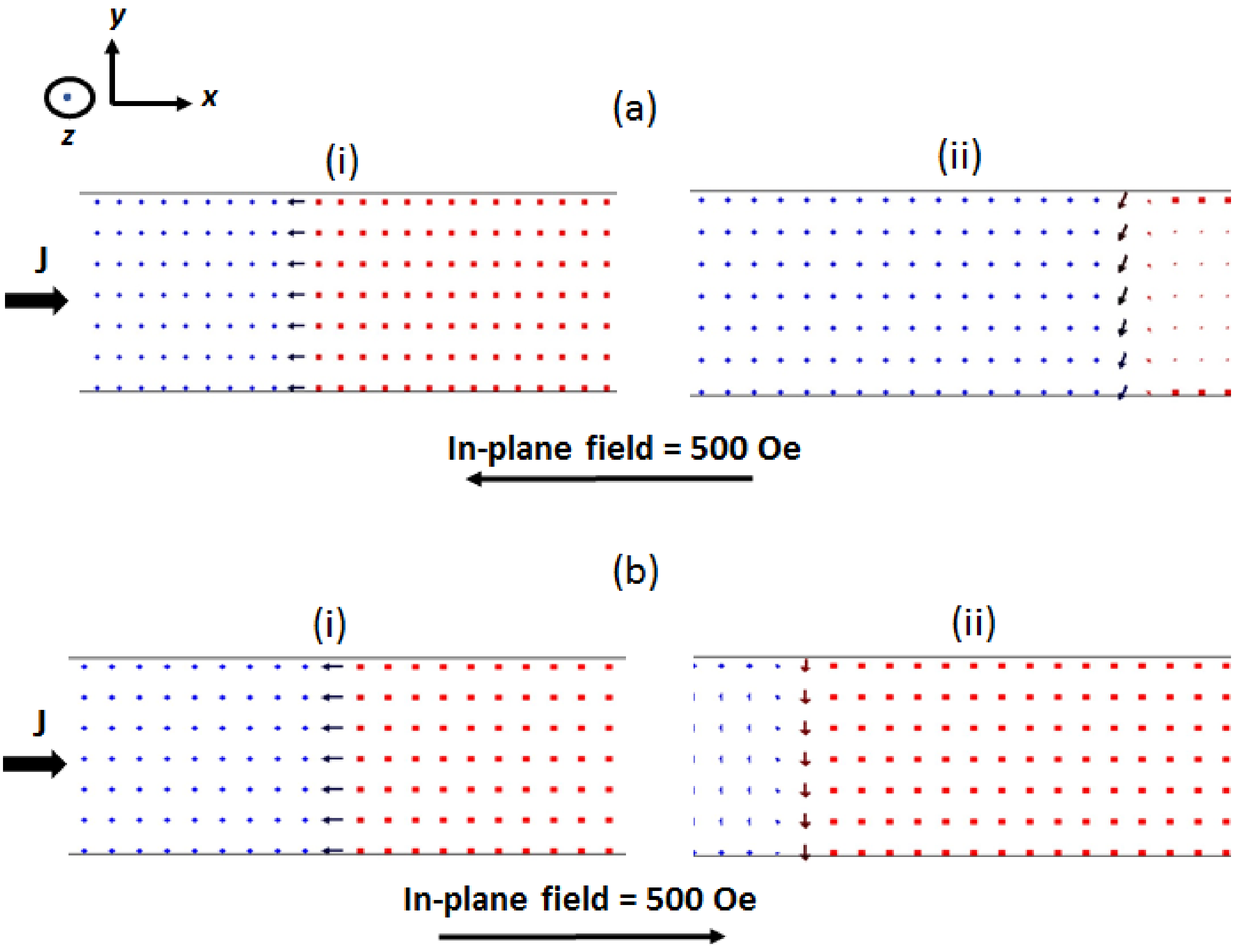

Figure S2. Micromagnetic simulations to show the effect of in-plane field on DW motion. Current is applied along +*x*-direction. DMI, D = 0.1 mJ/m^2 (a) Field = 500 Oe along −*x*-direction (i) Initial configuration (ii) Final configuration. (b) Field = 500 Oe along +*x*-direction (i) Initial configuration (ii) Final configuration.

S3. Domain wall velocities: Down-Up chirality

Figure S3 (a) shows the results of domain wall (DW) velocity with applied current density, the DW being nucleated in a down-up chirality. The results are similar to the ones shown in Figure 1 (c) with the difference being that magnetic field in the +*x*-direction now favours DW motion along the current flow and opposite is the case with magnetic field in the –*x*-direction. Only slight differences in the magnitude of the velocities were observed. The other observations are similar to the ones reported for Figure 1 (c). Figure S3 (b) shows the results when Ta thickness is increased to 3 nm and Figures S3 (c-d) show the comparison.

Figure S4 shows the plot when Pt spacer thickness is increased. The observations are similar to the ones reported in Figure 4 with the in-plane field direction reversed. Only slight differences in the magnitude of the velocities were observed.

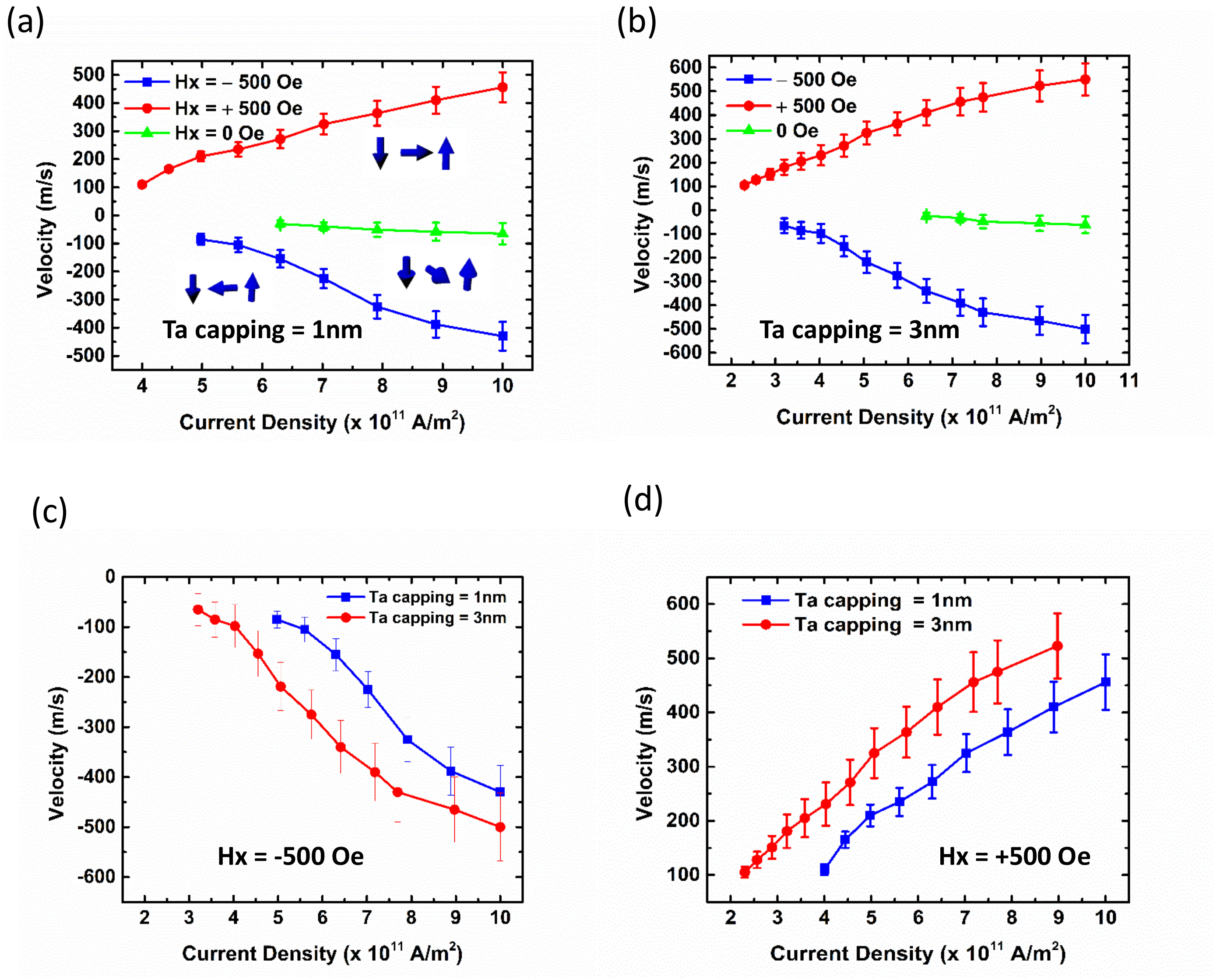

Figure S3. Down-up domain wall (DW) velocity versus current density in the presence of fixed magnetic field along and against *x*-direction (a) Ta capping thickness = 1nm. (b) Ta capping thickness = 3 nm. (c,d) Comparison of velocities for Ta thicknesses 1nm and 3nm respectively, (c) magnetic field along –*x*-direction (d) magnetic field along +*x*-direction.

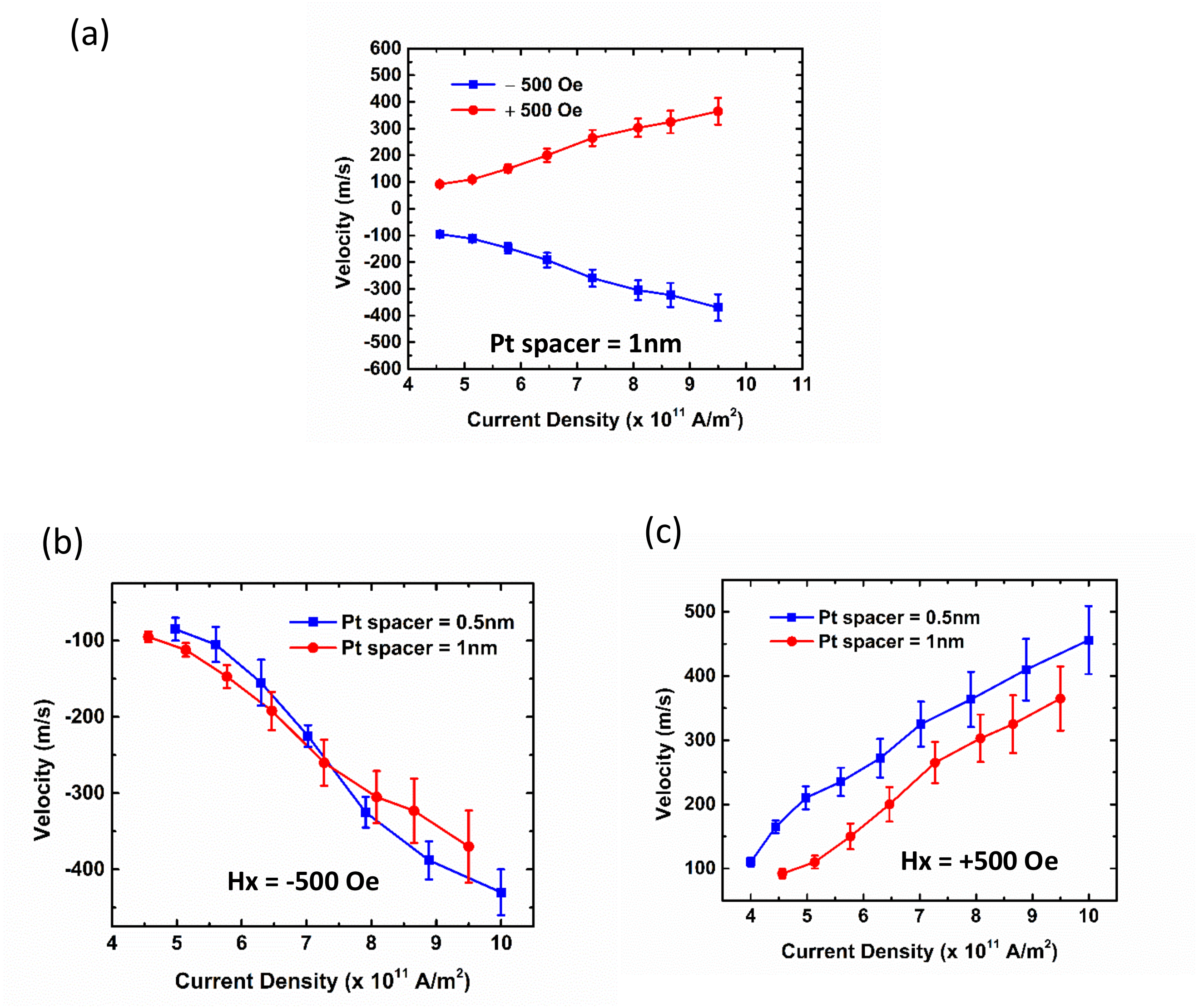

Figure S4. Down-up domain wall (DW) velocity versus current density in the presence of fixed magnetic field along and against *x*-direction (a) Pt spacer thickness = 1nm. (b-c) Comparison of velocities for Pt spacer thicknesses 0.5nm and 1nm respectively, (b) magnetic field along –*x*-direction (c) magnetic field along +*x*-direction.

S4. Estimation of DMI field for larger Pt spacer thickness

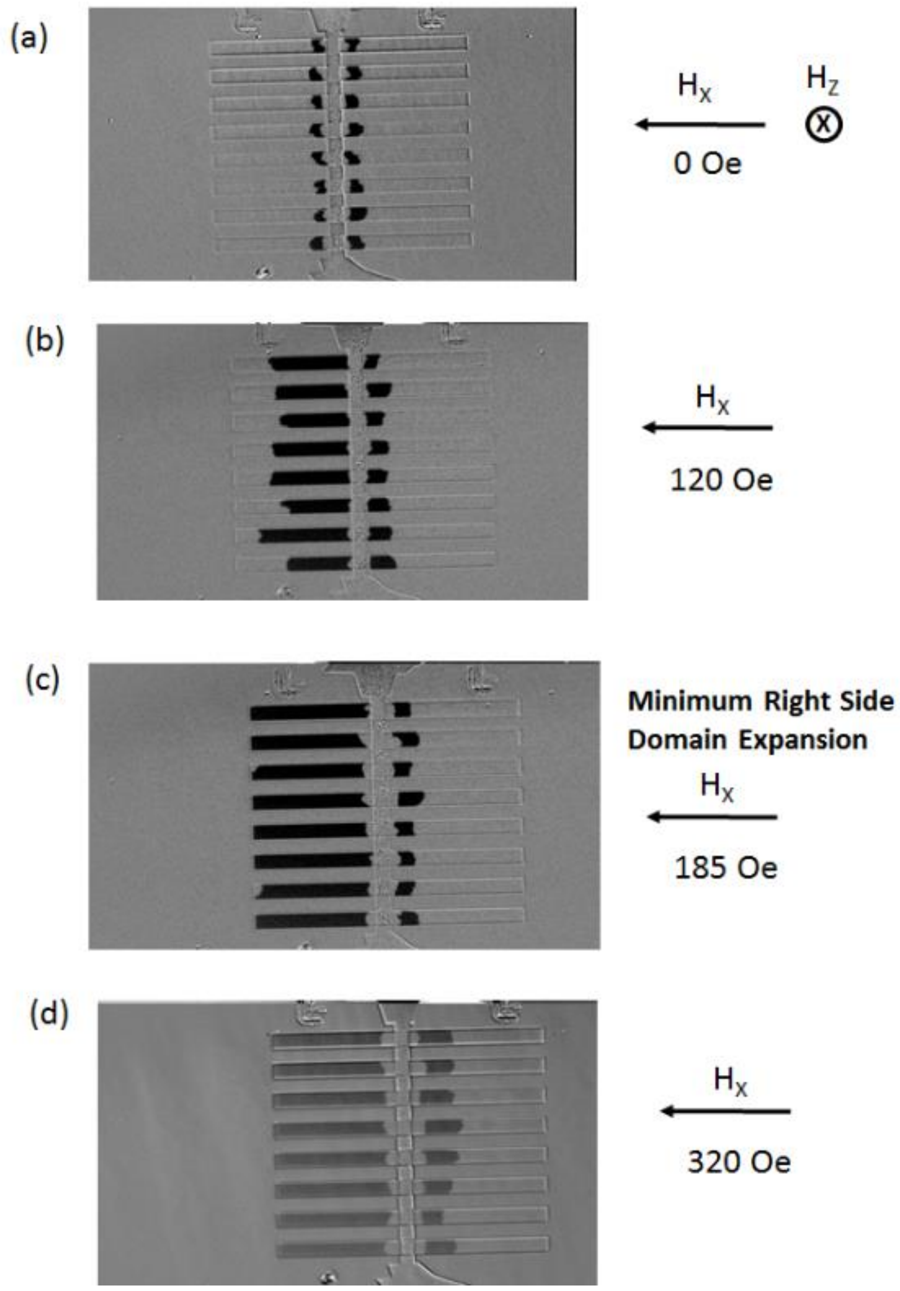

Figure S5. Kerr imaging to show DW creep in the presence of out-of-plane and in-plane magnetic field. The in-plane field opposes DMI field for DW propagating towards right and minimum displacement occurs at field equal to DMI field. Here H_{DMI} = 185-200 Oe.

Similar measurements as that reported in the manuscript were repeated for stack with Pt spacer as 1 nm thick. The stack used in the measurements was SiO_2/Ta (3)/Pt (3)/Co (0.7)/Pt (1)/Co (0.7)/Ta (1). Figure S5 above shows the Kerr imaging as in-plane magnetic field is gradually increased. The minimum displacement is obtained at around 185-200 Oe also plotted in Figure S6 below.

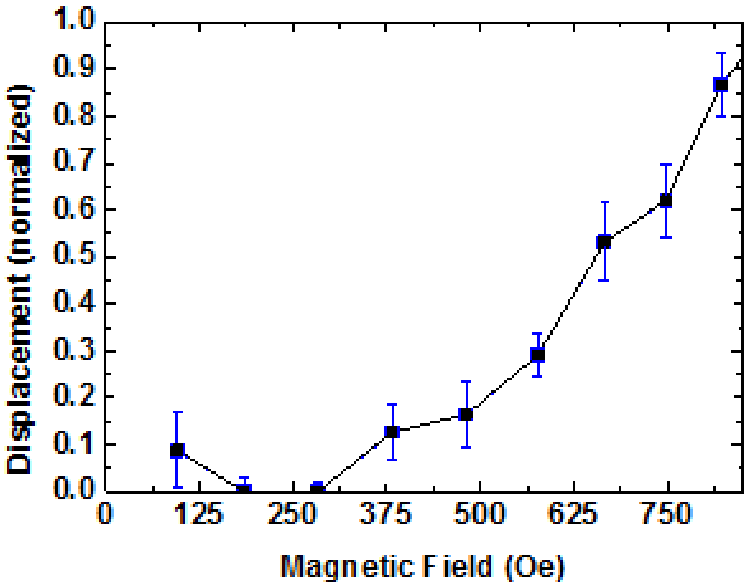

Figure S6. Domain wall displacement as a function of in-plane magnetic field. Minimum displacement corresponds to DMI field = 185-200 Oe.

Thus the H_{DMI} field for thicker Pt spacer thickness (= 1 nm) is roughly half of that when the Pt spacer thickness is less (= 0.5 nm).

S5. X-ray photoelectron spectroscopy of Co/Pt double stack

X-ray photoelectron spectroscopy (XPS) measurements were performed on the Co/Pt double stack with the following stack composition: Ta (3)/Pt (3)/Co (0.7)/Pt (0.5)/Co (0.7)/Ta (3)/Pt (1), where thicknesses are in nm. Figure S7 (a-c) shows the spectra of Pt, Ta and Co respectively with their respective binding energies. Clearly TaOx is not present and CoO is not formed due to the absence of satellite at 786 eV. Figure S8 below shows the atomic concentration depth profile of the multilayer stack. The middle region Co/Pt/Co shows almost overlap between the Co layers, since the Pt thickness involved is very low (0.5 nm) which is less than sputter rate used in the XPS at 45° angle (0.51 nm/s). It can be masked by the neighboring strong Pt signal. Also the surface roughness could lead to reduction in signal strength. Moreover, we observe that Pt concentration keeps going higher from the middle portion of Co/Pt/Co indicating that Pt is indeed present. Since the measurement area is 200 µm × 200 µm, we cannot comment on the continuity of Pt from these measurements. The XPS sampling depth is less than 0.5-2nm for metal, in order to reduce its effect, the take off-angle is reduced to 10 degree in these measurements to reduce etch rate.

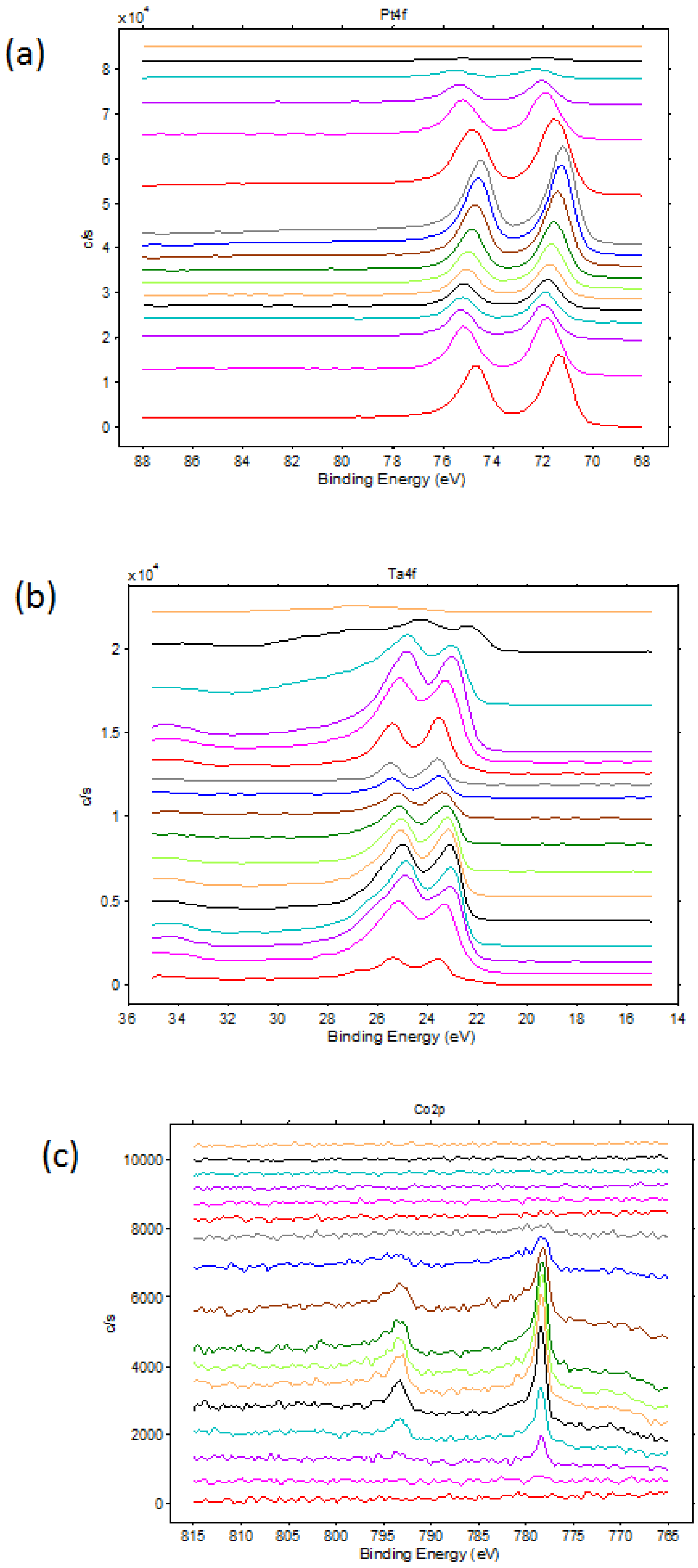

Figure S7. The spectrum of elements and their binding energies obtained using XPS, (a) Pt, (b) Ta and (c) Co

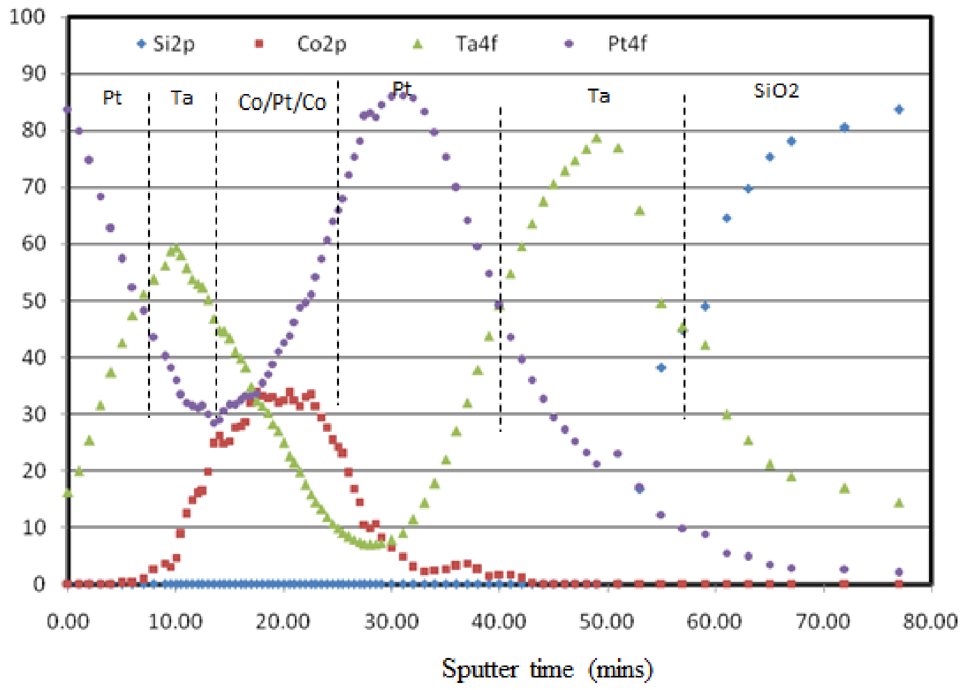

Figure S8. Atomic concentration of depth profile obtained by XPS measurements for the Co/Pt double stack

In order to compare DW dynamics in our proposed double stack with Pt/Co/Ta single layer stack, we deposited Pt/Co/Ta thin films on silicon substrate. A reduction in coercivity and PMA strength was observed on annealing the samples, reported by us.[3] This potentially due to diffusion of Ta into Co layer, as proposed by Woo *et al* through their x-ray photoelectron spectroscopy (XPS) results[4]. It is worth noting that the stack proposed by Woo *et al*, consisted of TaO_X as the capping layer to break the inversion symmetry. This layer is absent in our stack. There is little evidence to suggest enhancement in SHE strength with the presence of an oxide layer as it is not in direct contact with the ferromagnetic layer, unless there is diffusion of oxygen towards the ferromagnetic layer[5]. Moreover, the XPS results revealed a reduction in oxygen intermixing with Co layer at larger Ta layer thickness and increase in the Ta layer thickness showed enhanced SOT in the stack. In the stack proposed by us also, we cannot exclude the possibility of Co and Pt intermixing. However, recent report by Wells et al.[6]

suggests no significant change in magnetic properties or PMA degradation on account of Co and Pt intermixing. Hall cross geometry was fabricated and the DW driving measurements were repeated. However, random DW nucleations in the device was observed on passing an in-plane current, similar to the observations reported by us for Co/Ni stack[7]. Hence, comparatively Co/Pt double stack would give more advantage for DW driving study.

S6. Stray field of permanent bar magnet

The bar magnet used in our study is a permanent magnet cylindrical in shape. The magnetization is primarily along the horizontal direction. We have simulated the magnetic field lines using COMSOL tool. Figure S9 (a) below shows the field lines. The vertical component of the magnetic field is very weak near its axis. Figure S9 (b) is the magnified view with the device placed along the axis of the bar magnet. The stray field experienced by the device along the vertical direction is very less.

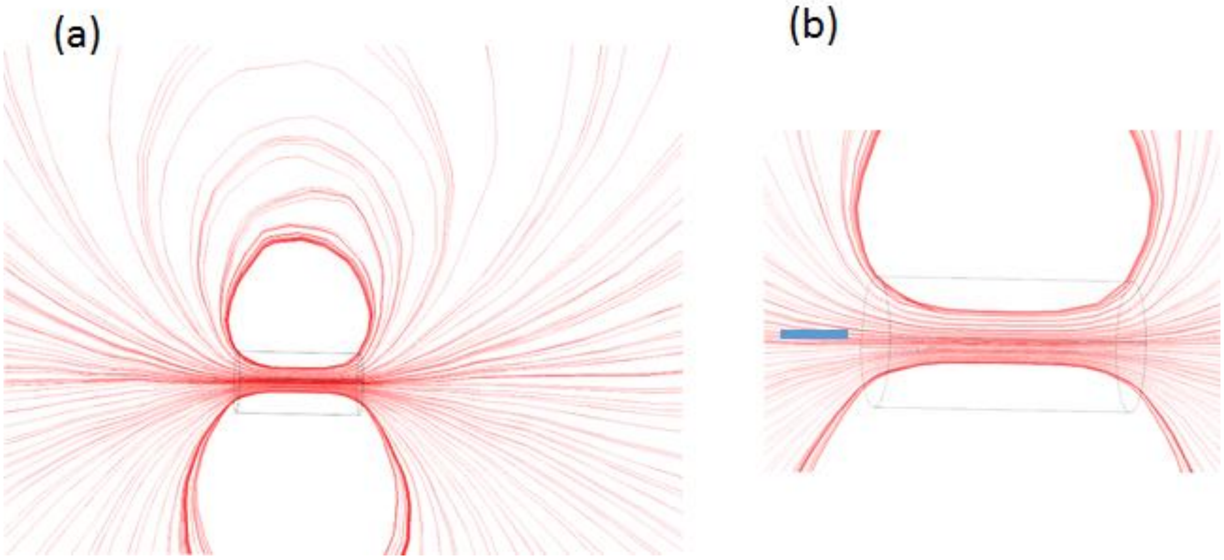

Figure S9. (a) Field lines due to a permanent cylindrical bar magnet. (b) The device, placed along the axis of the bar magnet experiences field along the horizontal direction, the stray field along the vertical direction is weak.

S7. Time of flight technique for domain wall velocity measurement

In the time of flight technique, a current pulse of known magnitude and pulse width, t_p, is applied across the nanowire to drive the DW. The DW can be detected at a known distance by sensing it presence, such as a Hall probe placed at a certain distance, d, from the point of DW injection. The DW would reach the Hall probe and a drop in Hall voltage would be detected via an oscilloscope after injecting n pulses of duration, t_p. This would give the DW velocity as $v = d/nt_p$. The error in such a technique can be minimized by reducing the Hall probe width. In this section we present some results taken from literature which use this technique for estimation of DW velocity.

Y. Yoshimura *et al.*[8] fabricated Hall cross structures of width 150 nm, and measured the DW velocity with respect to applied current density and magnetic field with a similar technique. Koyama *et al.*[9] from the same group as above have shown DW velocity measurements using the time-of-flight technique. Multiple current pulses of duration 10 ns were injected to drive the DW and a drop was observed at a certain critical current density. Recently, Yoshimura *et al.*[10] developed time-of-flight technique to estimate DW velocity for their Hall cross structures with respect to the out-of-plane field. In their work, the researchers applied a field less than the nucleation field and greater than DW driving field. Then a local Oersted field was generated by passing a current through a strip-line to nucleate DWs. As soon as the DW was nucleated it was driven by the existing driving field and detected as drop in Hall voltage at the 100nm wide Hall probe.

S8. Domain-wall velocity as a function of Ta capping thickness and relation to spin Hall angle

Figure S10 (a) shows the plot of DW velocity when Ta thickness is varied from 1 nm to 5 nm. The velocity gets saturated beyond thickness of 3 nm. Figure S10 (b) shows the plot of the spin Hall angle estimated for the same thickness ranges. The trend in spin Hall angle and the velocity is similar indicating the dependence of velocity on the SOT strength. The saturation is due to the limitation of spin diffusion length as mentioned in the manuscript.

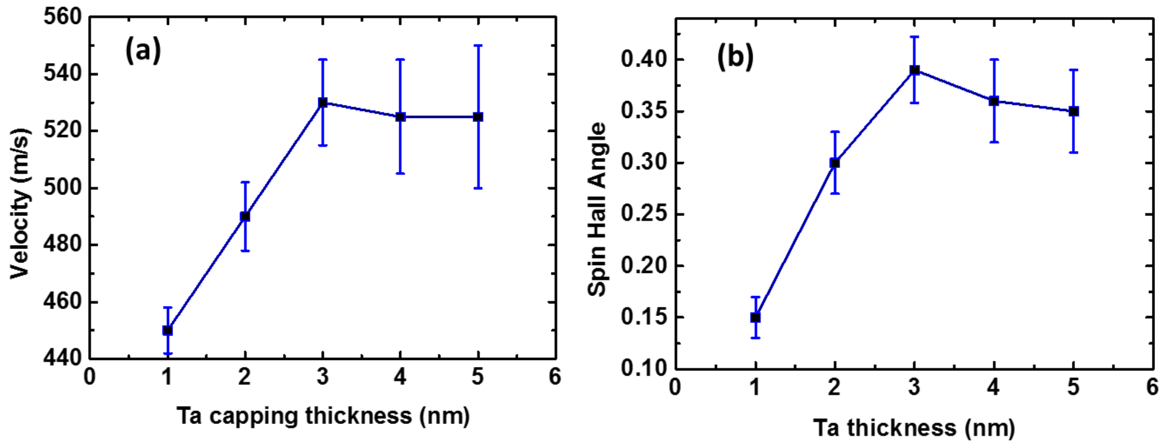

Figure S10 (a) DW velocity as a function of Ta capping thickness. (b) Spin Hall angle as a fuction of Ta capping thickness.

References

1 Vansteenkiste, A. & Van de Wiele, B. MUMAX: A new high-performance micromagnetic simulation tool. *J Magn Magn Mater* **323**, 2585-2591, doi:10.1016/j.jmmm.2011.05.037 (2011).

2 Vansteenkiste, A. *et al.* The design and verification of MuMax3. *Aip Adv* **4**, 107133, doi:10.1063/1.4899186 (2014).

3 Sethi, P., Krishnia, S., Li, S. H. & Lew, W. S. Modulation of spin-orbit torque efficiency by thickness control of heavy metal layers in Co/Pt multilayers. *J Magn Magn Mater* **426**, 497-503, doi:10.1016/j.jmmm.2016.11.130 (2017).

4 Woo, S., Mann, M., Tan, A. J., Caretta, L. & Beach, G. S. D. Enhanced spin-orbit torques in Pt/Co/Ta heterostructures. *Appl Phys Lett* **105**, doi:10.1063/1.4902529 (2014).

5 Qiu, X. *et al.* Spin-orbit-torque engineering via oxygen manipulation. *Nat Nanotechnol* **10**, 333-338, doi:10.1038/nnano.2015.18 (2015).

6 Wells, A. W. J., Shepley, P. M., Marrows, C. H. & Moore, T. A. Effect of interfacial intermixing on the Dzyaloshinskii-Moriya interaction in Pt/Co/Pt. *Phys Rev B* **95**, 054428, doi:10.1103/Physrevb.95.054428 (2017).

7 Sethi, P., Murapaka, C., Lim, G. J. & Lew, W. S. In-plane current induced domain wall nucleation and its stochasticity in perpendicular magnetic anisotropy Hall cross structures. *Appl Phys Lett* **107**, doi:10.1063/1.4935347 (2015).

8 Yoshimura, Y. *et al.* Current-Induced Domain Wall Motion in Perpendicularly Magnetized Co/Ni Nanowire under In-Plane Magnetic Fields. *Appl Phys Express* **5**, 063001, doi:10.1143/Apex.5.063001 (2012).

9 Koyama, T. *et al.* Current-Induced Magnetic Domain Wall Motion in a Co/Ni Nanowire with Structural Inversion Asymmetry. *Appl Phys Express* **6**, 033001, doi:10.7567/Apex.6.033001 (2013).

10 Yoshimura, Y. *et al.* Soliton-like magnetic domain wall motion induced by the interfacial Dzyaloshinskii-Moriya interaction. *Nat Phys*, doi:10.1038/nphys3535 (2015).
